# Parents of children with psychopathology: psychiatric problems and the association with their child’s problems

**DOI:** 10.1007/s00787-015-0813-2

**Published:** 2016-01-13

**Authors:** Christel M. Middeldorp, Laura W. Wesseldijk, James J. Hudziak, Frank C. Verhulst, Ramon J. L. Lindauer, Gwen C. Dieleman

**Affiliations:** 1 Department of Biological Psychology, Neuroscience Campus Amsterdam, VU University Amsterdam, Van der Boechorststraat 1, 1081 BT Amsterdam, The Netherlands; 2 Department of Child and Adolescent Psychiatry, GGZ inGeest/VU University Medical Center, Overschiestraat 57, 1062 HN Amsterdam, The Netherlands; 3 Department of Biological Psychology, EMGO+ Institute for Health and Care Research, VU University Amsterdam, Van der Boechorststraat 1, 1081 BT Amsterdam, The Netherlands; 4 Division of Human Genetics, Department of Psychiatry and Medicine, Center for Children, Youth and Families, University of Vermont, UHC Campus, Arnold 3, 1 South Prospect, Burlington, VT 05401 USA; 5 Department of Child and Adolescent Psychiatry/Psychology, Erasmus Medical Center Rotterdam, Wytemaweg 80, 3015 CN Rotterdam, The Netherlands; 6 Department of Child and Adolescent Psychiatry, De Bascule, Academic Center for Child and Adolescent Psychiatry, Meibergdreef 5, 1105 AZ Amsterdam ZO, The Netherlands

**Keywords:** Parental psychopathology, Internalizing symptoms, Externalizing symptoms, ADHD, Familial transmission

## Abstract

Knowledge is lacking regarding current psychopathology in parents whose children are evaluated in a psychiatric outpatient clinic. This especially accounts for fathers. We provide insight into the prevalence rates of parental psychopathology and the association with their offspring psychopathology by analyzing data on psychiatric problems collected in 701 mothers and 530 fathers of 757 referred children. Prevalence rates of parental psychopathology were based on (sub)clinical scores on the adult self report. Parent–offspring associations were investigated in multivariate analyses taking into account co-morbidity. Around 20 % of the parents had a (sub)clinical score on internalizing problems and around 10 % on attention deficit hyperactivity (ADH) problems. Prevalence rates did not differ between mothers and fathers. Parent–offspring associations did not differ between girls and boys. Maternal anxiety was associated with all offspring problem scores. In addition, maternal ADH problems were associated with offspring ADH problems. Paternal anxiety and ADH problems scores were specifically associated with offspring internalizing and externalizing problem scores, respectively. Associations with offspring psychopathology were of similar magnitude for mothers and fathers and were not influenced by spousal resemblance. Our study shows that both fathers and mothers are at increased risk for psychiatric problems at the time of a child’s evaluation and that their problems are equally associated with their offspring problems. The results emphasize the need to screen mothers as well as fathers for psychiatric problems. Specific treatment programs should be developed for these families in especially high need.

## Introduction

It is well established that psychiatric disorders run in families. Moreover, the increased risk for psychopathology in parents or children is not confined to the disorder of, respectively, the children or the parents, but also extends to other disorders [1, 2]. These findings were mostly observed using a lifetime history of psychiatric illness approach. However, since ongoing psychopathology in parents can influence the course and treatment outcome of psychopathology in children [3–7], knowledge about parental psychiatric problems at the time a child is suffering from a psychiatric disorder is also important.

So far, it has been shown that parents whose children are evaluated for psychiatric disorders at a child and adolescent psychiatric outpatient clinic, are at higher risk for internalizing problems and disorders, such as anxiety and depression. Prevalence rates range from 18 to 68 % [8–17]. Far less information is available on parental externalizing problems and disorders, such as attention deficit hyperactivity disorder and antisocial personality disorder, but the risk also seems increased [8–10, 12].

Information on paternal psychopathology is further lacking. In many of the former studies far fewer fathers than mothers were included and in four the data were restricted to mothers [9, 11, 12, 14]. Still, several studies have indicated that paternal psychopathology is also associated with offspring psychopathology [18]. In addition, paternal psychopathology may influence the association between maternal and offspring symptoms due to spousal resemblance. Kim-Cohen et al. [19] found that the association between maternal depression and offspring externalizing problems was diminished, although still significant, when paternal antisocial personality disorder was included in the analysis. In contrast, Marmorstein et al. [20] observed no attenuation of the effects of either major depression in mothers or antisocial personality in fathers on major depression and conduct disorder in their offspring.

Finally, the majority of these studies selected children with specific diagnoses, i.e., depression [9, 17], anxiety [8, 11], or ADHD/conduct disorder (CD) [10, 14–16]. Given that family studies clearly indicate that the associations between family members are not confined to the disorders of the probands, only an offspring population with a broad range of psychopathology provides good insight into the association between psychopathology of parents and children.

The current study provides prevalence rates of current psychiatric problems in mothers as well as fathers of children suffering from various psychiatric disorders. In addition, the associations between parental and offspring problems in these families are reported. Internalizing as well as externalizing problem scales were assessed in a large sample of parents at the time of the first appointment of their child in a child and adolescent psychiatric outpatient clinic. Similar problem scales were measured in parents and children. The associations between the parental and offspring problem scores were analyzed within and across the different syndrome scales and the analyses were performed separately for boys and girls. Finally, we investigated whether the maternal and paternal problems were independently associated with offspring problems or whether these associations were partly explained by spousal resemblance for psychiatric problems.

## Methods

### Participants

Data have been collected in three child and adolescent outpatient clinics in The Netherlands (two in Amsterdam and one in Rotterdam). In these clinics, parents already rated the children’s problems using the child behavior checklist (CBCL) [21] as part of the standard clinical procedure at the first assessment. Families with children aged between 6 and 18 years were included. In Amsterdam, first, a pilot study was carried out to examine how many parents are at risk for a psychiatric disorder at the moment of the first assessment of their child at a child and adolescent psychiatric outpatient clinic. Out of the 176 mothers and 122 fathers of 191 children that completed the adult self report (ASR) [22], 38.2 % of the mothers and 31.5 % of the fathers scored in the (sub)clinical range on at least one of the syndrome scales. Consequently, assessment of parental problems and, if necessary, further assessment and subsequent treatment, became the standard procedure. The total Amsterdam sample consists of 363 mothers and 235 fathers from 389 families, after exclusion of 35 families without consent for the use of the data for research and of 26 non-biological parents. The study was approved by the Central Ethics Committee on Research Involving Human Subjects of the VU University Medical Centre, Amsterdam. In the Rotterdam outpatient clinic, data were collected as part of the standard clinical procedure from the start. If parents reported (sub)clinical problems, psychopathology was further assessed, and, if necessary, parents were referred to adult mental health services. Data were available for 338 mothers and 295 fathers from 368 families, after exclusion from 29 non-biological parents. Family response rates, i.e., the percentage of families in which at least one parental questionnaire was completed, were 70 % in the Amsterdam and 60 % in the Rotterdam samples. In total, data were analyzed from 701 mothers and 530 fathers from 757 families.

The most common diagnoses in children in the Amsterdam and Rotterdam cohort were ADHD (40 and 30 %), autism spectrum disorders (18 and 26 %), behavioral disorders (11 and 9 %), anxiety disorders (17 and 24 %) and depressive disorders (12 and 4 %). These disorders are not mutually exclusive, i.e., children can have more than one diagnosis. The parental scores in the Amsterdam and Rotterdam cohort did not differ for the ASR syndrome scales (Wesseldijk et al. submitted). The parents from the Amsterdam cohort were on average 3 years older and higher educated (Wesseldijk et al. submitted). This latter difference is in line with the known regional differences in educational achievement in the Netherlands, with people in Rotterdam having on average a lower education than in Amsterdam [23]. The children in the Amsterdam cohort were also on average 1.5 years older than the children in the Rotterdam dam cohort. Parental and offspring age and parental education were included as covariates in the analyses.

### Measures

Demographical information on age, sex and educational achievement was obtained from the questionnaire. Educational achievement was analyzed in three categories, i.e., low (at most lower secondary schooling), intermediate (at most higher secondary schooling) and high educational achievement.

Behavioral and emotional problems in parents and children were measured with the age-appropriate version of the questionnaires belonging to the Achenbach system of empirically based assessment (ASEBA), i.e., the CBCL [21] and the ASR [24]. In both generations, the DSM-oriented syndrome scales were analyzed. For the children, the depressive, anxiety, attention deficit/hyperactivity (ADH), oppositional defiant and conduct problem scales were included and for the parents depressive, anxiety, avoidant personality, ADH, and antisocial personality problem scales.

For both the CBCL and the ASR, thresholds for (sub)clinical scores for each sex are provided in the manual. The thresholds for the subclinical and clinical scores reflect the 93rd and 97th percentile, respectively, in men and women of the general population.

### Analyses

To investigate potential response bias, we analyzed whether the children’s scores differed according to the participation of the parents. We made four groups of children for boys and girls: (1) none of the parents participated, (2) both parents participated, (3) father participated, and (4) mother participated. Differences in mean syndrome scale scores between the four groups were analyzed with an ANOVA.

Based on the thresholds provided in the manual, the prevalence rates of fathers and mothers with (sub)clinical scores were calculated for each scale. These prevalence rates give an indication of how many parents are likely to suffer from clinically relevant psychiatric symptoms.

**Fig. 1:**
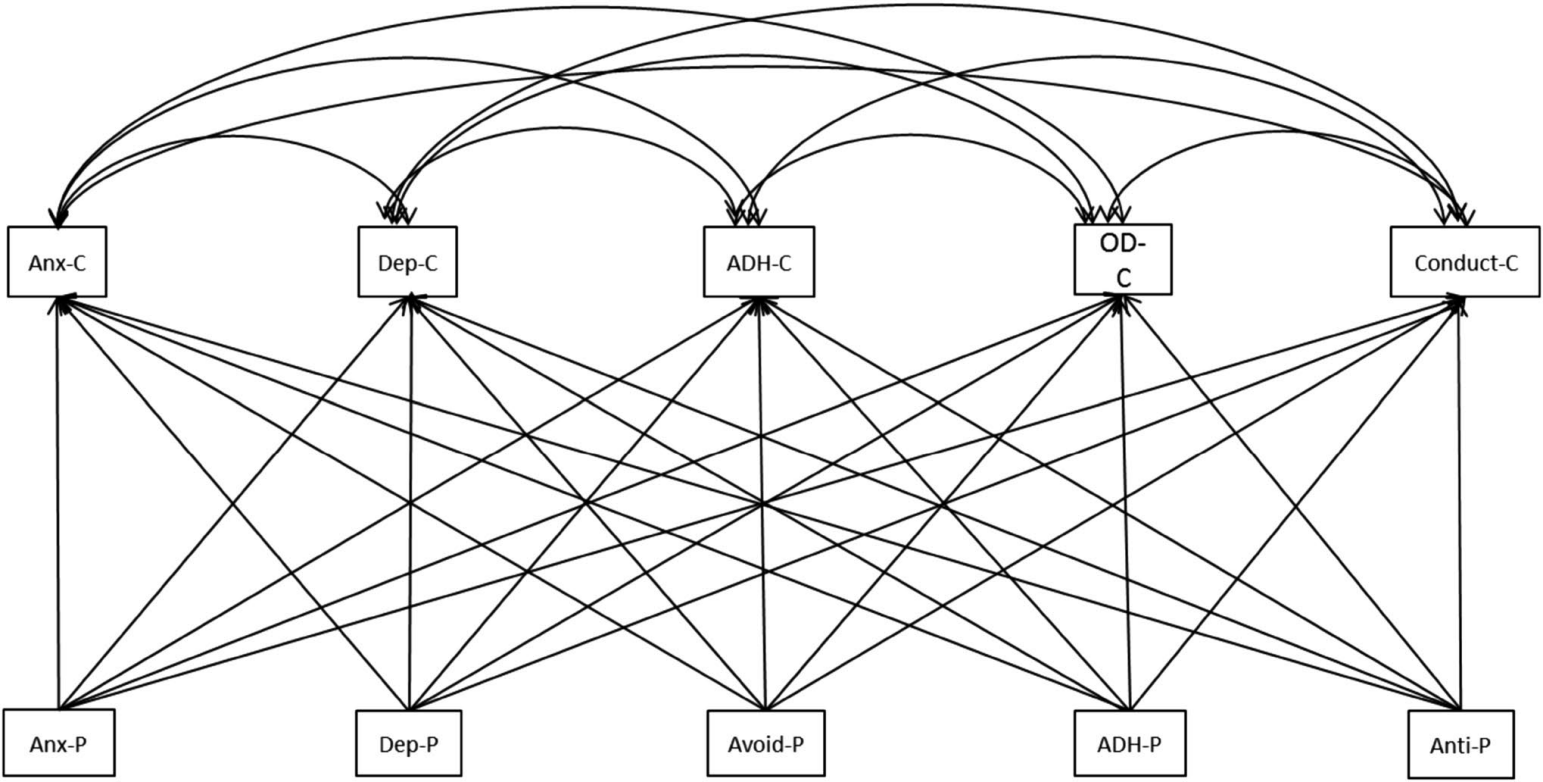
The multivariate model: The childhood problems scores (C) are correlated. The parental (P) problem scores predict each childhood problem score. The model was analyzed for maternal and paternal problem scores separately. *ADH* attention deficit/hyper activity, *Anti* antisocial personality, *Anx* anxiety, *Avoid* avoidant personality, *Dep* depressive, *OD* oppositional defiant problems

All other analyses were carried out on the continuous scores of the DSM-oriented scales so that all available information on individual variation was used. Pearson’s correlations between parental and offspring problem scores were calculated within and across syndrome scales. This was followed by a multivariate multi-group analysis in Mplus in which the problem scores in boys and girls were predicted by the maternal problem scores or the paternal problem scores (see Fig. 1). First, the analyses were performed separately for girls and boys. Next, it was tested whether there were sex differences by constraining the beta’s to be equal over the sexes. In these analyses, we made optimal use of the available parental data since measures from families in which only one parent participated were also included. However, these analyses do not take into account spousal resemblance for psychopathology, which has been detected in the current sample with correlations varying between 0.13 and 0.30 within and across the syndrome scales (Wesseldijk et al. submitted). To investigate whether the effects of maternal and paternal psychopathology can be explained by spousal resemblance, we also tested a model in which both the maternal and paternal scores were included that had a marginally significant effect (*p* < 0.10) in the first analyses. If these effects remain similar in the order of magnitude and significance, spousal resemblance does not explain the association with childhood psychopathology. Age from parents and children and parental education were included as covariates.

## Results

### Descriptives of participants and prevalence rates of parental psychiatric problems

Children in our sample scored higher than children from a population-based sample [25] and their scores follow the well-known pattern of girls scoring higher on internalizing problems and boys on externalizing problems (Table 1). Table 2 shows the mean CBCL scores for the four possible response patterns in the families: (1) both parents, (2) mother, (3) father or (4) none of the parents completed the ASR. Children whose parents did not participate did not generally score higher than children of whom both parents participated. Only two of the ten analyses showed a significant between group difference in mean scores, i.e., for depressive and conduct problems in girls. This appeared to be due to the group of girls of whom only the mother completed the ASR. These girls scored higher than the girls in the other three groups. Further analyses comparing the families in which one or two parents completed the ASR revealed that factors associated with psychopathology were more prevalent in the families in which only the mother completed the ASR. These were more often broken or single parent families (65 % compared to 37 % of the families in which the father completed the ASR and to 15 % in the families in which both parents completed the ASR) and the level of education of the mother was lower (43 % in the lowest category compared to 26 and 21 %).

**Table 1:** Mean (SD) age and scores on the DSM-oriented syndrome scales in girls and boys (top) and mean age (SD), education (%) and number of parents (%) with a score in the (sub)clinical range (bottom)

	Girls (*n* = 296)	Boys (*n* = 375)
Age	11.7 (3.4)	10.5 (3.2)
Depression	7.1 (4.6)	5.4 (4.0)
Anxiety	4.5 (3.1)	3.6 (2.7)
ADH	5.4 (3.7)	7.2 (3.6)
Oppositional defiant	3.7 (2.8)	4.2 (2.5)
Conduct	3.5 (4.2)	4.4 (4.2)

	Mothers (*n* = 701)	Fathers (*n* = 530)
Age	41.9 (6.4)	44.9 (6.7)
Parental education: low/middle/high	26/58/16	27/57/19
Depression	107 (15.3)	67 (12.7)
Anxiety	52 (7.4)	32 (6.1)
Avoidant personality	59 (8.4)	55 (10.4)
ADH	71 (10.2)	49 (9.3)
Antisocial personality	36 (5.1)	34 (6.4)
Total	174 (24.8)	127 (24)

**Table 2:** Mean problem scores (SD) in boys and girls of whom (1) no parents participated (No), (2) both parents participated (M + F), (3) only father participated (F), (4) only mother participated (M)

	Girls				Boys			
	No (114)	M + F (205)	F (13)	M (85)	No (167)	M + F (264)	F (24)	M (98)
Dep	6.3 (4.7)	6.7 (4.4)	6.7 (4.3)	8.6 (4.8)*	5.6 (4.1)	5.3 (3.9)	5.5 (4.3)	5.6 (4.1)
Anx	3.7 (2.8)	4.6 (3.1)	4.4 (2.4)	4.5 (3.0)	3.7 (2.7)	3.8 (2.7)	2.7 (2.1)	3.3 (2.6)
ADH	5.5 (3.8)	5.2 (3.6)	5.2 (4.1)	6.0 (3.9)	7.4 (3.7)	7.3 (3.6)	6.3 (3.7)	7.5 (3.6)
OD	3.4 (2.7)	3.6 (2.6)	2.9 (1.9)	4.3 (3.1)	4.2 (2.8)	4.1 (2.6)	4.1 (2.5)	4.6 (2.6)
Conduct	3.7 (4.4)	2.9 (3.6)	3.0 (3.5)	5.1 (5.6)*	4.9 (4.9)	4.2 (4.1)	5.5 (5.7)	4.8 (4.1)

*ADH* attention deficit/hyperactivity problems, *Anx* anxiety problems, *Dep* depressive problems, *OD* oppositional defiant problems

* *p* < 0.005

Around 25 % of the parents had a (sub)clinical score on one of the analyzed scales (Table 2). A (sub)clinical score on depressive problems was most prevalent with 13 % of the fathers and 15 % of the mothers scoring above the threshold (Table 1). These rates are clearly higher than the rate of 7 % (sub)clinical scores in the general population on which the cut-offs are based [24]. The percentages are also higher for avoidant personality and ADH problems. In total, 20 % of the mothers and 18 % of the fathers had a (sub)clinical score on the internalizing scales anxiety, depressive or avoidant personality problems. There were no significant differences between mothers and fathers.

### Parent–offspring associations

Table 3 shows the correlations between the parental and offspring problem scores, separately for girls and boys and mothers and fathers. Almost all parent–offspring correlations were significant and ranged between 0.15 and 0.25. A notable exception was anxiety problem scores in girls which were neither associated with paternal ADH nor with maternal and paternal antisocial personality problem scores. This was also seen in boys, but only for maternal and not for paternal problem scores. Another notable exception was conduct problem scores in boys, which were not associated with any of the paternal internalizing problem scores.

**Table 3:** Correlations between parental and offspring problem scores (M = maternal and P = paternal)

	Mother					Father				
	Dep	Anx	Avoid	ADH	Anti	Dep	Anx	Avoid	ADH	Anti
Dep.										
Girls	**0**.**27**	**0**.**28**	**0**.**25**	**0**.**17**	**0**.**12**	**0**.**30**	**0**.**27**	**0**.**12**	**0**.**23**	**0**.**20**
Boys	**0**.**22**	**0**.**30**	**0**.**22**	**0**.**20**	**0**.**18**	**0**.**31**	**0**.**36**	**0**.**25**	**0**.**27**	**0**.**19**
Anx.										
Girls	**0**.**16**	**0**.**26**	**0**.**28**	**0**.**18**	0.08	0.13	**0**.**21**	**0**.**14**	0.04	−0.03
Boys	**0**.**13**	**0**.**23**	**0**.**19**	0.10	0.01	**0**.**25**	**0**.**33**	**0**.**18**	**0**.**25**	**0**.**16**
ADH										
Girls	**0**.**18**	**0**.**21**	**0**.**18**	**0**.**25**	**0**.**14**	**0**.**26**	0.11	**0**.**15**	**0**.**32**	**0**.**17**
Boys	**0**.**15**	**0**.**19**	**0**.**14**	**0**.**27**	**0**.**13**	**0**.**12**	**0**.**13**	0.05	**0**.**20**	0.03
OD										
Girls	**0**.**23**	**0**.**23**	**0**.**18**	**0**.**23**	**0**.**20**	**0**.**32**	**0**.**22**	**0**.**20**	**0**.**32**	**0**.**21**
Boys	**0**.**16**	**0**.**21**	**0**.**15**	**0**.**16**	**0**.**16**	**0**.**14**	**0**.**17**	0.08	**0**.**25**	**0**.**15**
Conduct										
Girls	**0**.**15**	**0**.**17**	0.10	**0**.**19**	**0**.**15**	**0**.**26**	0.10	**0**.**18**	**0**.**22**	**0**.**18**
Boys	**0**.**18**	**0**.**21**	0.10	**0**.**17**	**0**.**17**	0.10	0.10	0.10	**0**.**16**	**0**.**13**

In bold the significant correlations (*p* < 0.05)

*ADH* attention deficit/hyperactivity, *Anti* antisocial personality, *Anx* anxiety, *Avoid* avoidant personality, *Dep* depressive, *OD* oppositional defiant problems

Subsequently, we performed the multivariate analyses predicting the offspring scores by maternal or paternal problem scores. Constraining the regression coefficients to be equal for boys and girls revealed no significant differences in the effects of the maternal (*p* = 0.99) and paternal scores (*p* = 0.90) on childhood psychopathology.

The results of the multivariate analyses indicate that the significant correlations found in the univariate analyses are mainly due to the high with-in person correlations for the problem scores. It becomes clear from Table 4 that in the analyses of the maternal problem scores, anxiety was associated with all offspring psychopathology with larger effect sizes for childhood anxiety and depression (~0.20) than for the externalizing problem scores (~0.15). In addition, maternal ADH was associated with offspring ADH with an effect size of 0.20. In the analyses of the paternal problem scores, anxiety was also associated with childhood internalizing psychopathology (effect sizes ~0.20), but not with externalizing psychopathology. Paternal ADH was associated with childhood ADH and OD (effect sizes ~0.20). There were no significant associations with childhood conduct problems.

**Table 4:** Standardized regression coefficients for the multivariate analyses with childhood psychopathology predicted by maternal (top) or paternal problem scores (bottom)

	Childhood dep			Childhood anx			Childhood ADH			Childhood OD			Childhood conduct		
	*β*	SE	*p*	*β*	SE	*p*	*β*	SE	*p*	*β*	SE	*p*	*β*	SE	*p*
Mother															
Dep	−0.04	0.07	0.58	−**0**.**15**	**0**.**07**	**0**.**04**	−0.09	0.07	0.15	−0.02	0.07	0.79	0.004	0.07	0.95
Anx	**0**.**21**	**0**.**06**	<**0**.**001**	**0**.**25**	**0**.**06**	<**0**.**001**	**0**.**15**	**0**.**06**	**0**.**02**	**0**.**15**	**0**.**06**	**0**.**01**	**0**.**14**	**0**.**06**	**0**.**02**
Avoid	**0**.**13**	**0**.**06**	**0**.**02**	**0**.**22**	**0**.**05**	<**0**.**001**	0.01	0.05	0.84	0.01	0.05	0.86	−0.07	0.06	0.23
ADH	0.04	0.05	0.38	0.04	0.05	0.43	**0**.**24**	**0**.**05**	<**0**.**001**	0.07	0.05	0.16	0.08	0.06	0.16
Anti	0.01	0.05	0.88	−0.09	0.05	0.06	−0.02	0.05	0.61	0.09	0.05	0.06	0.08	0.05	0.07
Father															
Dep	0.14	0.08	0.09	−0.02	0.08	0.84	0.11	0.07	0.16	0.05	0.08	0.50	0.09	0.08	0.27
Anx	**0**.**18**	**0**.**06**	**0**.**01**	**0**.**25**	**0**.**07**	**0**.**001**	−0.004	0.07	0.95	0.06	0.06	0.34	−0.04	0.06	0.56
Avoid	−0.02	0.05	0.63	0.05	0.06	0.44	−0.04	0.06	0.50	−0.03	0.06	0.62	0.03	0.07	0.63
ADH	0.04	0.07	0.55	0.04	0.07	0.55	**0**.**23**	**0**.**06**	<**0**.**001**	**0**.**20**	**0**.**06**	**0**.**001**	0.09	0.08	0.25
Anti	0.05	0.07	0.45	−0.06	0.07	0.43	−0.09	0.05	0.10	0.02	0.06	0.67	0.06	0.06	0.31

Bold are the regression coefficients with a *p* value below 0.05

*ADH* attention deficit/hyper activity, *Anti* antisocial personality, *Anx* anxiety, *Avoid* avoidant personality, *Dep* depressive, *OD* oppositional defiant problems

The multivariate analyses including the maternal and paternal problems scores with a *p* value below 0.10 did not show substantial differences in the results. This indicates that the predictions as found in the separate analyses of the maternal and paternal problem scores not due to spousal resemblance in psychiatric problems.

## Discussion

This study shows that parents whose children suffer from a variety of psychopathology have a higher risk of experiencing depressive, avoidant personality, and ADH problems than adults in the general population. In contrast to what is found in epidemiological studies in the general population, prevalence rates did not differ between sexes, i.e., mothers and fathers were equally affected with internalizing and ADH problems. Regarding the parent–offspring associations in problem scores at the time of a child’s diagnostic evaluation in a child and adolescent psychiatric outpatient clinic, the similarities over the sexes are striking. The associations are not different for boys and girls and maternal problems are as associated with offspring problems as paternal problems. The only difference is that maternal anxiety is associated with all childhood problem scores while paternal anxiety is only associated with childhood internalizing problems. Childhood externalizing problems are associated with paternal ADH problems and childhood ADH problems are associated with maternal ADH problems.

Overall, our results indicate the usefulness of screening parents on psychopathology when their child is evaluated at a child and adolescent psychiatric outpatient clinic. For mothers, this screening should focus on internalizing problems, irrespective of the offspring psychopathology, and on ADH problems when the child experiences ADH problems. For fathers, the focus should be on internalizing problems when children are presented with internalizing problems and on ADH problems when children are presented with externalizing problems. Treatment programs should be developed specifically focused on these multiple affected families. The similarities in the results of mothers and fathers, both for the prevalence rates and for the associations with the offspring problems indicate the need to include fathers in such a screening and subsequent treatment.

### Prevalence rates

The higher rates of (sub)clinical scores on the internalizing syndrome scales in fathers and mothers (20 %) are in line with other studies investigating current symptoms at the time of the first assessment of a child in an outpatient psychiatric clinic, although the percentages observed in the current study are somewhat lower compared to the previous studies [8–17]. In addition, we found that parents of children with psychopathology have a higher prevalence rate of (sub)clinical ADH problems, as suggested by one other study in mothers [16]. The equal rates of (sub)clinical scores on antisocial personality problems in comparison to the general population are in contrast to one earlier study investigating parental antisocial personality [10]. The lack of increased antisocial personality problems and the somewhat lower percentages of internalizing problems may be related to the sample of children in which the data were collected. Most of the previous studies selected children based on one or two diagnoses, i.e., depression, or ADHD and/or conduct disorder. In the study showing higher rates of antisocial personality disorder in parents, for example, this was only true for children with conduct disorder with or without ADHD and not for children with only ADHD. Only two studies included children with a broad range of psychopathology, just as in the current study [12, 13]. The prevalence rate for parental internalizing disorders was comparable to ours in one study (18 %), but a lot higher (57 %) in the other study. Part of the difference with the latter study could be due to the demographic characteristics of the included parents. The sample of the study with similar estimates to ours is more comparable regarding socio-economic status and age of the parents. Future studies collecting data in families of children evaluated in a general child and adolescent outpatient clinic may shed more light on these large differences and identify which parents are especially at risk for psychopathology.

### Parent–offspring associations in problem scores

The specificity observed in the father-offspring associations and the lack of associations with maternal externalizing problem scores may seem in contrast to large family studies which indicate that children of parents with psychopathology are not only at risk for the disorder of the parent, but for a broad range of disorders and vice versa [1, 2]. One of these studies did not take the frequent co-morbidity of psychiatric disorders into account [1] which can result in significant correlations across disorders as illustrated by the many significant correlations in the univariate analyses in the current study. Three other important differences are that these family studies (1) investigated lifetime disorders in (2) population-based samples and (3) did not stratify their analyses by sex [1, 2]. The differences with the current results are therefore difficult to interpret.

The strength of the predictions of the offspring problem scores was similar for the paternal and the maternal problem scores (effect sizes ~0.20) and were not attenuated in the analyses including the problem scores of both parents simultaneously. This indicates that psychopathology in fathers and mothers are equally associated with offspring psychopathology agreeing with previous findings as summarized in a review on the influence of paternal psychopathology on their offspring [18]. By simultaneously analyzing the predictions of offspring scores by maternal and paternal problem scores, we also showed that the contributions of the parents are independent of each other, thus not due to spousal resemblance which is also present in the current study population [correlations within and across syndrome scales varying between 0.13 and 0.30 (Wesseldijk et al. submitted manuscript)]. This has already been suggested before by studies focusing on depression and/or antisocial personality [19, 20, 26]. These results underline the importance of involving fathers in research.

### Limitations

A few limitations should be kept in mind. Although this sample has one of the largest numbers of fathers included, the participation rate in fathers was still lower than in mothers. Our non-response analyses suggested that families in which only the mothers participated were exposed to less favorable circumstances than the families in which both parents or only the father participated. This pattern of partly non-response in the fathers could have led to an underestimation of the prevalence rates for psychopathology in fathers. It should still be kept in mind that part of these families are single parent families who are known to be at higher risk for adverse events.

It could be considered a limitation that all problem scores were based on parental ratings, either of the child (CBCL) or of the parents (ASR), and not on a more objective measure, such as clinical diagnosis. However, both the CBCL and the ASR problem scores are associated with psychiatric diagnoses. This has been repeatedly demonstrated for the CBCL (see, e.g., [27, 28]). Further, a study in a subsample of the parents with a (sub)clinical score has shown that 71 and 74 % of, respectively, these fathers and mothers have a lifetime psychiatric mood or anxiety disorder according to the composite international diagnostic interview (CIDI) [29] or ADHD according to the Conners’ Adult ADHD Rating Scales (CAARS) [30]. The advantage of analyzing continuous scores is that they capture more information on the individual variation in the presence of psychiatric problems. This signifies that, for example, subclinical comorbid symptoms that do not fulfill the criteria for a DSM-IV diagnosis are reflected in a higher than average problem score. A strength is that the CBCL and the ASR are specifically designed to measure similar constructs over ages, which make them particularly suitable for studying associations between children and parents.

The disadvantage of having one parental measure, instead of two, of the child’s psychopathology is that it has been suggested that parental problems can influence the ratings of their child’s problems. In 75 % of the cases, the questionnaire was rated by the mother. Studies investigating the influence of parental mood symptoms on the assessment of their children yielded discrepant results (see [31] for an overview). Overall, it is probably safest to say that parental mood symptoms may increase the parental report of children’s problems, but only to a small extent. Our finding that the association between paternal psychopathology and offspring psychopathology is of similar magnitude as the association with maternal psychopathology suggests that shared measurement variance is not of major influence to our results.

It is not possible to draw any conclusions about the mechanisms underlying the association between parental and offspring problems. Twin studies have shown that differences in the susceptibility for psychiatric disorders or traits are substantially explained by genetic factors with an average heritability of 46 % [32]. For childhood phenotypes, in contrast to adult phenotypes, an effect of the shared familial environment has also been found with estimates ranging from 10 to 30 % in meta-analyses of several measures of internalizing and externalizing problems [33]. For ADHD and related traits, shared environmental influences are consistently found to be absent [33]. Thus, in general, transmission of psychopathology from parents to children can go through genetic as well as environmental factors. Other study designs, such as adoption studies or extended twin designs (including parents of the twins or children of the twins) can further disentangle to what extent the association between parental and offspring psychopathology is due to genetic or environmental effects. A review of children-of twins studies indicates that both effects can play a role, depending on the phenotype under investigation [34].

Our findings also do not imply a direction of effect, i.e., the association between parental and offspring problems is not necessarily entirely explained by the transmission of problems from parents to children. It has been shown that a successful depression treatment in mothers also results in a decrease of psychiatric problems in children [7]. However, the reverse has also been found, mothers whose daughters were treated for depression also showed an improvement of their depressive symptoms [35]. It should be noted that still a large group of children and mothers had continuing symptoms despite treatment of the mothers or daughters. Longitudinal studies are needed to elucidate in which families it is sufficient to only treat the admitted patient and in which families, all affected members should be treated at once.

### Future steps

This study confirms that part of the families seen in a child and adolescent psychiatric outpatient clinic is in especially high need as not only the child, but also one or both parents are affected. Future studies, involving both mothers and fathers, are warranted to further investigate the associations between parent and offspring problems, not only concurrently, but also longitudinally, and to develop specific treatment programs for these families. The question also arises what the prevalence rates are of psychiatric problems in children whose parents are evaluated in a psychiatric outpatient clinic. There have been several family studies investigating offspring psychopathology in parents with psychopathology. However, these mainly focused on lifetime disorders in the offspring [36]. Knowledge is lacking regarding current psychopathology at the time a parent is evaluated for a psychiatric disorder. A study similar to ours, but performed in an adult psychiatric outpatient clinic and focusing on the current problems of the children can indicate for which disorders these children are at risk and inform further treatment studies.
